# Diabetes knowledge and utilization of healthcare services among patients with type 2 diabetes mellitus in Dhaka, Bangladesh

**DOI:** 10.1186/s12913-017-2542-3

**Published:** 2017-08-22

**Authors:** Md. Kaoser Bin Siddique, Sheikh Mohammed Shariful Islam, Palash Chandra Banik, Lal B. Rawal

**Affiliations:** 1 James P. Grant School of Public Health (JPGSPH), BRAC University, Dhaka, Bangladesh; 2 PhysioCare, Physiotherapy, Rehab & Research Center (PPRRC), Dhaka, Bangladesh; 3 Non-Communicable Diseases Initiative, icddr,b, Dhaka, Bangladesh; 4 The George Institute for Global Health, University of Sydney, Sydney, Australia; 5 Institute for Physical Activity and Nutrition (IPAN), Deakin University, Melbourne, Australia; 6 Department of Noncommunicable Diseases, Bangladesh University of Health Sciences (BUHS), Dhaka, Bangladesh; 7 Health Systems and Population Studies Division, icddr,b, Dhaka, Bangladesh

**Keywords:** Diabetes, Healthcare services, Health systems, Non-communicable diseases, Risk factors

## Abstract

**Background:**

Diabetes is a significant global public health concern. Poor knowledge of disease and healthcare utilization is associated with worse health outcomes, leading to increasing burden of diabetes in many developing countries. This study aimed to determine diabetes related knowledge and factors affecting utilization of healthcare services among patients with type 2 diabetes mellitus in Bangladesh.

**Methods:**

This analytical study was conducted among 318 patients with type 2 diabetes (T2DM) attending two large tertiary hospitals in Dhaka, Bangladesh between August 2014 and January 2015. Interviewer assisted semi-structured survey questionnaire was used to collect data on diabetes knowledge (measured by a validated Likert scale) and self-reported utilization of service for diabetes. Univariate and bivariate analyses were conducted to determine the factors associated with diabetes knowledge and healthcare utilization.

**Results:**

The mean (±SD) age of participants was 52 (±10) years. Majority of the participants were females (58%) and urban residents (74%). Almost two-third (66%) of the participants had an average level of knowledge of T2DM. One-fifth (21%) of the participants had poor knowledge which was significantly associated with gender (*P* < 0.002), education (*P* < 0 .001) and income (*P* < 0.001). The median travel and waiting time at the facility was 30 and 45 min respectively. More than one-third (37%) of the participants checked their blood glucose monthly. Most patients were satisfied regarding the family (55%) and hospital (67%) support.

**Conclusion:**

T2DM patients had average knowledge of diabetes which might affect the utilization of healthcare services for diabetes management. Innovations in increasing diabetes knowledge and health behavior change are recommended specially for females, those with lower education and less income.

**Electronic supplementary material:**

The online version of this article (doi:10.1186/s12913-017-2542-3) contains supplementary material, which is available to authorized users.

## Background

Non-communicable diseases are emerging public health problems in the rapidly changing world, particularly for low-and-middle income countries [1, 2]. Diabetes, cardiovascular diseases (CVDs), chronic respiratory diseases, and cancer are the major NCDs with highest burden of morbidity and mortality globally; accounting for 7.9 million deaths annually [3]. Almost 80% of the deaths worldwide are due to the diabetes and cardiovascular diseases [4]. An approximately 415 million adults were living with diabetes in 2015, about 80% of them were living in low-and-middle income countries and 46% of them were undiagnosed [5].

The prevalence of diabetes is increasing in Bangladesh in both urban and rural areas in recent years [6]. A recent study reported that majority adults with type 2 diabetes in Bangladesh have uncontrolled diabetes with a high prevalence of risk factors attributing to early development of complications [7]. Another study shows that diabetes patients in Bangladesh had limited knowledge on the causes, management and risk factors for diabetes [8]. Diabetes increases morbidity and mortality, impairs quality of life and thereby contributes to increased healthcare costs and burden in Bangladesh [9, 10].

Previous studies have reported poor access to healthcare and services in Bangladesh [11]. Several factors limit the utilization of desired diabetic control services for patients such as low socio-economic condition, knowledge and perception towards diabetes [12]. Utilization of diabetic services might also be affected by income, health literacy, depression, and competing demands, including those related to family dynamics and support are important for managing diabetes conditions effectively [12]. Improving access to utilization of healthcare services for diabetes is essential to improve diabetes management and prevent complications. Also, patient’s knowledge about disease influence health seeking behavior and it is essential to know the knowledge of the patients for better health planning. However, information on access to healthcare, knowledge and utilization of services for diabetes are scares in Bangladesh. This study aimed to determine diabetes related knowledge and factors affecting utilization of healthcare services in patients with type 2 diabetes mellitus attending tertiary level hospitals in Dhaka city.

## Methods

### Study design and site

This analytical study was conducted at the Bangladesh Institute of Health Science (BIHS) hospital and Dhaka Medical College Hospital (DMCH) in Dhaka, Bangladesh between August 2014 and January 2015. BIHS is a tertiary level private hospital affiliated with the Diabetes Association of Bangladesh. DMCH is one of the largest public hospital in Dhaka. The outpatient department of BIHS and DMCH serves a large number patients from Dhaka city and surrounding regions.

### Sampling strategy

Adult patients (aged >30 years old) with type-2 diabetes mellitus visiting the BIHS and DMCH for receiving outdoor diabetes services were recruited through non-probability purposive sampling. Eligible participants were referred by the attending physicians at the outpatient department. Patients with mental illness and severely ill requiring immediate hospitalization were excluded.

### Data collection tool development and procedure

Semi-structured survey questionnaire was used to collect data. We reviewed relevant literature and tools to develop the study questionnaire. The final questionnaire comprised 42 questions which divided into four parts: socio-demography (11), knowledge (9), perception (6) and service utilization (16). The English version of the questionnaire was translated into Bangla and back translated into English. The questionnaire was tested at the outpatient department of another tertiary hospital: Bangladesh Institute of Research and Rehabilitation for Diabetes, Endocrine and Metabolic Disorders (BIRDEM) hospital among 40 patients with type 2 diabetes mellitus to check the suitability of the tools. After, pretesting, necessary modification and rephrasing was done to develop the final questionnaire (Additional file 1: Table S1). Research Assistants were trained on ethical issues and administering the questionnaires.

All eligible participants attending the T2DM outdoor service were approached and offered to participate in this study for an exit interview after consultation with a physician. After describing the purpose of this study those who willingly agreed to sign the consent form were selected as study participant. Interviews were conducted face-to-face at the patient’s waiting room to ensure the privacy. Each interview lasted around 20-30 min and sufficient time was given to participants to minimize recall bias and assumption.

Diabetes knowledge was assessed using a Likert scale knowledge questionnaire. The knowledge questionnaire was divided into four sections: knowledge on risk factors, prevention, control and complication of diabetes. Each part had multiple responses. For each correct answer 1 (one) and wrong answer 0 (zero) points were given, and the mean was calculated through compute variable option. Respondent those who scored “*<Mean − 1 (SD)”* counted as poor knowledge, “*Mean ± 1 (SD)”* average and “*>Mean + 1 (SD)”* counted as a good knowledge. The knowledge tools were used and validated in a previous study in Bangladesh and other countries [13–15]. A conceptual framework was developed for understanding healthcare utilization (Additional file 1: Figure S1).

### Data entry, sample size and analysis

All questionnaires were checked manually after the interviews for missing data and inconsistencies which were cross cheeked with repeating the question. Internal consistency was checked among the interviewer. Data were entered into Microsoft Excel sheet and after cleaning, transferred into the Statistical Package for Social Sciences (SPSS) software program version 20.0 (Armonk, New York, USA) for analysis. Data were verified through internal consistency checking and comparing with other findings. Assuming, the number of doctor visit for utilization of diabetic services is 50%, at 95% confidence interval actual estimated sample size of this study was 384. Continuous data were presented as mean ± standard deviation (SD) or median (inter quartile range) and categorical data were presented as number and percentage. Categorical data were analyzed by Pearson’s chi square test, as appropriate. Univariate and multivariate models were performed to access factors associated with diabetes knowledge and healthcare service utilization for diabetes. A *p*-value < 0.05 was considered statistically significant.

#### Ethical issue

Ethics approval of the study was obtained from the Ethics Review Committee of the James P. Grant School of Public Health, BRAC University. Written informed consent was taken from all participants and they were informed about the purpose of the study.

## Results

### General characteristics

The mean ± SD age of the participants was 52 ± 10 years, more than half (58.0%) of the respondents were females. Only 12% respondents were illiterate and majority (74%) were from urban areas (Table 1). The majority of the patients reported that they had knowledge (self-reported yes/no) on diabetes risk factors, prevention, control and complications, which were 83%, 81%, 95% and 91% respectively (data not presented in the table).

**Table 1:** General characteristics of the respondents (*n* = 318)

Variable	Category	Male (*n* = 134) 42%	Female (*n* = 184) 58%	Total (*n* = 318) (100%)
Study site	*BIHS*	122 (44.0)	158 (56.0)	280 (88.0)
	*DMCH*	12 (32.0)	26 (68.0)	38 (12.0)
Age	*30-39*	14 (37.0)	24 (63.0)	38 (12.0)
	*40-49*	21 (27.0)	58 (73.0)	79 (25.0)
	*50-59*	42 (42.0)	58 (58.0)	100 (31.0)
	*≥60*	57 (56.0)	44 (44.0)	101 (32.0)
Marital status	*Never married*	4 (57.0)	3 (43.0)	7 (2.2)
	*Married*	130 (43.0)	173 (57.0)	303 (95.3)
	*Others*	0 (0.0)	8 (100.0)	8 (2.5)
Religion	*Islam*	130 (42.0)	182 (58.0)	312 (98.0)
	*Hindu*	4 (67.0)	2 (33.0)	6 (2.0)
Education	*No formal education*	8 (21.0)	30 (79.0)	38 (12.0)
	*Primary education*	25 (24.0)	78 (76.0)	103 (32.0)
	*Secondary education*	47 (44.0)	61 (56.0)	108 (34.0)
	*College and above*	54 (78.0)	15 (22.0)	69 (22.0)
Occupation	*Service*	52 (96.0)	2 (4.0)	54 (17.0)
	*Business*	32 (100.0)	0 (0.0)	32 (10.0)
	*Laborer*	5 (71.0)	2 (29.0)	7 (2.0)
	*Farming*	4 (80.0)	1 (20.0)	5 (2.0)
	*Housewife*	0 (0.0)	176 (100.0)	176 (55.0)
	*Retired*	38 (93.0)	3 (7.0)	41 (13.8)
	*Others*	3 (100.0)	0 (0.0)	3 (1.0)
Household member	*≤ 4*	58 (40.0)	87 (60.0)	145 (46.0)
	*>4*	76 (44.0)	97 (56.0)	173 (54.0)
Monthly family Income (*n* = 317), BDT	*<10,000*	18 (35.0)	34 (65.0)	52 (16.0)
	*10,000-29,999*	57 (39.0)	89 (61.0)	146 (46.0)
	*30,000-59,999*	41 (49.0)	42 (51.0)	83 (26.0)
Mean = 38,262	*60,000-89,999*	6 (55.0)	5 (45.0)	11 (3.0)
Median = 25,000	*≥90,000*	12 (46.0)	14 (54.0)	26 (8.0)
Monthly family expenditure, BDT	*<10,000*	21 (36.0)	37 (64.0)	58 (18.0)
	*10,000-29,999*	63 (41.0)	92 (59.0)	155 (49.0)
	*30,000-59,999*	38 (48.0)	42 (53.0)	80 (25.0)
Mean = 31,662	*60,000-89,999*	4 (44.0)	5 (56.0)	9 (3.0)
Median = 25,000	*≥90,000*	8 (50.0)	8 (50.0)	16 (5.0)
Residency of patients	*Urban*	97 (41.0)	137 (59.0)	234 (74.0)
	*Rural*	19 (49.0)	20 (51.0)	39 (12.0)
	*Peri Urban*	18 (40.0)	27 (60.0)	45 (14.0)

BDT Bangladeshi Taka (1 USD = 80 BDT, 2015)

### Knowledge & perception regarding diabetes prevention and management

The highest number of patients (43%) reported that genetic factors were responsible for the development of T2DM, while others mentioned obesity, physical inactivity and food habits (Table 2). Almost all of those risk factors showed statistically significant difference with socio-demographic factors (Table 2).

**Table 2:** Knowledge of Risk Factors, Prevention, Control and Complications of T2DM (*n* = 318)

	Risk Factors				Prevention		Control		Complications	
	Genetic	Obesity	Less PA	Balanced Diet	Increased PA	Balanced Diet	Increasing PA	Balanced Diet	Eye Problems	Kidney Problems
Frequency (%)	137 (43)	79 (25)	86 (27)	93 (29)	188 (59)	157 (49)	229 (72)	224 (70)	170 (54)	195 (61)
Gender	0.453	0.022*	0.001*	0.651	0.001*	0.075	0.704	0.005*	0.010*	0.018*
Age	0.245	0.918	0.302	0.624	0.115	0.842	0.316	0.975	0.836	0.005*
Education	0.049*	0.309	0.000*	0.010*	0.008*	0.006*	0.000*	0.351	0.009*	0.006*
Family Income	0.066	0.020*	0.036*	0.245	0.048*	0.001*	0.001*	0.209	0.098	0.030*

*Note:* Chi square test has been done

*values are significant (p < 0 .05)

*PA*, physical activity

In response to preventive measures, increasing physical activity and balanced diet dominated other responses, which was 59% and 49% respectively, followed by reducing weight (16%) and regular check-up (13%). Also, 7 % patients reported other prevention strategies: Treating other diseases, stop smoking etc. (data not presented in table or graph). Almost all those prevention strategies showed statistically significant difference with socio-demographic factors. A statistically significant relationship was present between level of education and increasing physical activity (*P* < 0.0001) (Table 2).

A great majority of the patients reported increased physical activity (72%) and reduced carbohydrate intake (70%) as first line diabetes control measure. Additionally, reducing weight and adherence to physician prescription accounted for 12% and 18% respectively. (Data not shown in table or figure).

The most frequent diabetes related complications reported by the participants were kidney problems (61.3%) and eye problems (53.5%), followed by neurological problems (17%) and cardiovascular problems (15%) (Table 2). Other diabetes complications mentioned by the patients were: uncontrolled diabetes, foot problems, dental problems, liver damage, which accounted for less than 5 %. Kidney problems showed statistically significant relationship with all the socio-economic status (SES) indicators (gender, age, education and income; *P* value <0.005, <0.006, <0.018 and <0.030), and others complication showed relationship with at least one SES indicators (Table 2 and Additional file 1: Table S2).

**Fig. 1:**
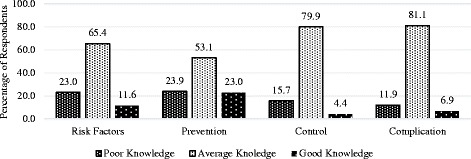
Level of Knowledge on Risk Factor, Prevention, Control and Complication of Diabetes (*n* = 318)

**Fig. 2:**
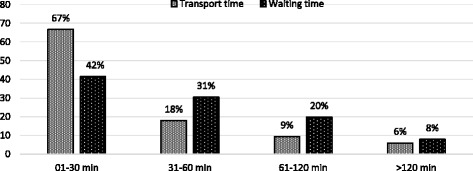
Duration of time spent for transport and waiting for diabetic service (*n* = 318)

Good knowledge on risk factors, prevention, control and complication of diabetes has been reported (Fig. 1). After compiling all four knowledge domains the frequency of *Good, Average* and *Poor* knowledge was 13%, 66% and 21% respectively. (data not shown in the graph). The duration of time spent for transport and waiting are presented in Fig. 2.

Table 3 portrayed that male responded scored better than female for good diabetes knowledge. Similarly, the frequency of good knowledge was higher in younger age groups (<60 years) compared to their older counterparts (>60 years). The level of knowledge about diabetes was gradually found better with increase in education level which was nearly six times higher than illiterate patients. There was statistically significant relationship between education and diabetes knowledge (*P* < 0 .001). Patients with less family income (<10,000 Bangladeshi Taka (BDT)/per month) portrayed significantly *poor knowledge (p < 0.001)*. (Table 3)*.*

**Table 3:** Relationship between SES and Knowledge about Diabetes (*n* = 318)

	Socio-demographic variables	Knowledge (%)			Statistical Indices
		Good	Average	Poor	
Gender	*Male*	27 (66)	86 (41)	21 (32)	χ^2=12.505^
	*Female*	14 (34)	125 (59)	45 (68)	df = 2
					*P* = 0.002
Age	*30-39*	13 (32)	27 (13)	5 (8)	χ^2=4.792^
	*40-49*	14 (34)	56 (27)	15 (23)	df = 6
	*50-59*	8 (20)	67 (32)	19 (29)	*P* = 0.571
	*≥60*	6 (15)	61 (29)	27 (41)	
Education	*No formal education*	2 (5)	17 (8)	19 (29)	χ^2=49.007^
	*Primary*	7 (17)	71 (34)	25 (38)	df = 6
	*Secondary*	11 (27)	82 (39)	15 (23)	*P* < 0.0001
	*College & above*	21 (51)	41 (19)	7 (11)	
Occupation	*Service*	10 (19)	38 (70)	6 (11)	χ^2=25.312^
	*Business*	8 (25)	19 (59)	5 (16)	df = 12
	*Laborer*	0 (0)	6 (86)	1 (14)	*P* = 0.013
	*Farming*	0 (0)	3 (60)	2 (40)	
	*Housewife*	43 (24)	120 (68)	13 (7)	
	*Retired*	10 (24)	24 (59)	7 (17)	
	*Others*	0 (0)	1 (33.3)	2 (66.7)	
Family Income	*< 10,000 BDT*	0 (0)	10 (5)	16 (24)	χ^2=40.712^
	*10,000-29,999 BDT*	16 (39)	95 (45)	28 (42)	df = 8
	*30,000-59,000 BDT*	12 (29)	76 (36)	15 (23)	*P* < 0.0001
	*60,000-89,000 BDT*	8 (20)	13 (6)	3 (5)	
	*≥ 90,000 BDT*	5 (12)	17 (8)	4 (6)	
Residence	*Urban*	32 (78)	157 (74)	45 (68)	χ^2=4.613^
	*Rural*	4 (10)	22 (10)	13 (20)	df = 4
	*Semi-urban*	5 (12)	32 (15)	8 (12)	*P* = .329

BDT Bangladeshi Taka (1 USD = 80 BDT, 2015)

The half of the rural patients scored average on knowledge. On the other hand, around two-third number of urban and semi-urban patients showed average knowledge, 67% and 71% respectively (Table 3).

Regarding perception about diabetes, mixed types of perception has been observed. One-third (31%) of the respondents perceived that diabetes is a result of excessive intake of sugar and 14% could not mention anything. Patients perception about diabetes showed statistically significant relationship with education except for perception that diabetes causes deaths (P 0.7) (Table 4). The frequency of doctor visit is presented in Table 5.

**Table 4:** Patients’ Perception towards Diabetes & Relationship with Education (*n* = 318)

Diabetes perception	True (%)	False (%)	Don’t know (%)	Statistical indices (χ^2^)
				Education
*Diabetes is a simple disease*	75 (23.6)	224 (70.4)	19 (6.0)	*P < 0* .018*
*Diabetes is a disease of rich patients*	76 (23.9)	232 (73)	10 (3.1)	P < 0.0001*
*Diabetes causes excessive intake of sugar*	98 (30.8)	177 (55.7)	43 (13.5)	P < 0 .006*
*Diabetes causes will of god*	224 (70.4)	70 (22)	24 (7.5)	P < 0 .004*
*Diabetes causes death*	282 (88.7)	20 (6.3)	16 (5.0)	*P < 0* .703
*Diabetes can be cured completely*	75 (23.6)	226 (71.1)	17 (5.3)	P < 0 .0001*

Note: *Tabulated values are significant (p < .05); not significant (p > .05)* Education (Those who completed secondary or higher education)

**Table 5:** Frequency of doctor visit in a year and in a quarter (3 months)

Frequency of doctor or health center visit	In a Year (%)	In 3 Months (%)	Total
*Once or less*	24 (7.5)	134 (42.1)	318 (100)
*2-4 times*	106 (33.3)	162 (50.9)	318 (100)
*5-6 times*	90 (28.3)	15 (4.7)	318 (100)
*7-12 times*	82 (25.8)	6 (1.9)	318 (100)
*>12 times*	16 (5.0)	1 (.3)	318 (100)

### Access & utilization of diabetes service

**Fig. 3:**
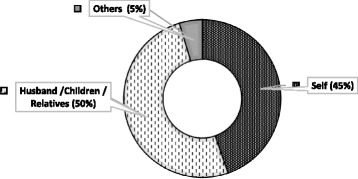
Responsible person for bearing the treatment cost (*n* = 318)

**Fig. 4:**
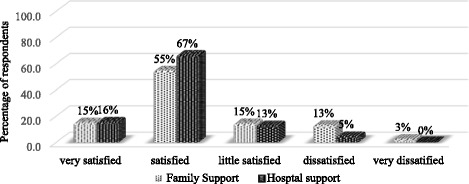
Patients’ satisfaction with family and hospital support (*n* = 318)

The median travel and waiting time at the facility was 30 and 45 min respectively. Similarly, the median travel cost and cost of blood sugar measurement were 30 and 120 BDT respectively and majority of people used public transport to visit hospital (data not shown). Persons responsible for bearing the treatment for for the participants are shown in Fig. 3. More than one-third of the patients (38%) measured their blood sugar once in a month either at home or at the hospital and 37% checked their blood sugar levels at least once in 3 months. Only 3 % tested blood sugar once in a year and nearly 2 % never checked their blood (Table 6). In most of the cases (50%) diabetic treatment costs were carried out by husband or children or relatives, followed by respondent themselves (45%). More than half of the patients were satisfied regarding the family (55%) and hospital (67%) support, while only 13% and 5% participants expressed dissatisfaction with the family and hospital support respectively. Only 3% of the participants were very dissatisfied with the family support and none with the hospital services (Fig. 4).

**Table 6:** Frequency and percentage of Blood Sugar Monitoring (*n* = 318)

Frequency of blood sugar monitoring	Number (%)
Several times in a day	6 (2)
Daily	01 (.3)
Weekly	58 (18)
Monthly	120 (38)
2-3 months after	119 (37)
Yearly	09 (3)
Never	05 (2)

## Discussion

This study shows that majority of patients with T2DM have average level of knowledge on diabetes risk factors, treatment, complication and prevention which is similar to previous studies in Bangladesh and India that showed moderate knowledge on diabetes among T2DM patients [13, 14]. Furthermore, a study in Singapore on general population showed similar results [16]. A study conducted in Aga Khan University Hospital found more than 50% diabetic patients have poor knowledge on symptom, treatment and complication which was almost three times higher than the findings obtained from this study (18%). Similarly, sub-optimal level of knowledge was found among semi-urban Omani population where only 55% patients knew about diabetic complications [17].

Patients’ level of education, occupation, income was associated with the level of knowledge on risk factors, prevention, control and complication of diabetes. Several studies also reported similar results that level of education has strong influence on knowledge [17–19]. However, a study by Mehrotra and colleagues reported no definite relationship between knowledge and occupation, but positive impact of education on overall knowledge levels [20]. According to Powell, Hill, & Clancy, (2007) for managing diabetes, health education can play an important role which is also determining factors for improving diabetic management through behavior modification [21, 22].

In our study genetic, obesity, physical inactivity and food habits were identified as major risk factors of diabetes and almost all of those risk factors had relationship with sex, occupation, monthly family income and expenditure of patients, which congruently matched with the result of a study by Al Shafaee et al. (2008) [17]. A systematic review of 14 studies on diabetes risk factors in Bangladesh also found these risk factors [23]. The majority of patients in our study agreed that diabetes can be prevented through balanced diet and increased physical activity which is in line with another study that showed education level as the most significant predictor of knowledge regarding risk factors, complications and the prevention of diabetes [17]. A study conducted at hospital outdoor setting in India showed that dietary modification (75%) and exercises (51%) can control diabetes in line with our findings [24]. Moreover, a recent study on general population in Bangladesh reported controlling diet (about 50%) is beneficial to control diabetes [25]. Depression was a risk factor for diabetes in previous studies in Bangladesh [26, 27], but not reported in our study.

In this study, 25% and 27% participants mentioned about obesity and lack of physical activity as risk factors for type 2 diabetes, which is similar to a previous study in Oman showing 29.5% and 20.8% participants reporting obesity and physical inactivity as diabetes risk factors [17]. A qualitative study among 12 diabetes patients in Bangladesh also reported that low-adherence to physical activity, diet and physician advice were problematic for the diabetes patients in Bangladesh [28].

Majority of patients (66%) in our study demonstrated *average knowledge* and only 13% showed *good knowledge* on diabetes, which is congruently matched with a study in similar setting by Saleh et al. (2012), where patients with T2DM reported *Good, Average and Poor knowledge*, 16%, 66% and 18% respectively [14]. A study by Islam et al. reported that Overall, 45.6% participants had good, 37.7% moderate and 16.7% poor knowledge on diabetes, which is higher than our participants [8]. However, many studies from developed and developing countries found that diabetic patient’s general knowledge on diabetes is *poor* [24, 29].

Although most of the patients knew that diabetes is a complex and fatal disease, 70% of the participants believed that diabetes is caused as a will of God. About one-third of patients perceived that excessive intake of sugar causes diabetes which is similar to another study in Bangladesh by Shariful Islam et al. (2015) [8]. Two separate studies in Oman and United Arab Emirates, found similar trend where high consumption of dietary sugar (about 60%) were reported as a major cause for developing diabetes [17, 29]. Therefore, we might assume that there is a significant knowledge gap between risk perception and knowledge on actual cause of diabetes among the diabetic patients.

Our study shows that women have less access to education (79% illiteracy), knowledge on diabetes (47% vs. 33%) and which was associated with patient’s personal income, satisfaction to family support and number of doctor visit. The utilization of diabetic service was influenced by demographic, socio-economic and educational factors which were supported by numerous studies in developed and developing countries [30]. On the other hand, educational qualification does not influence the patients to visit diabetic services reported by a hospital based study in Bangladesh [31]. Many studies from developed and developing countries reported that women generally enjoy less health care facility [32].

With regards to doctor visit, it is encouraging to reveal that most of the respondent visited doctor or any health care center 2-12 times in a year or at least once in a 3 months which is similar to a study in Australia, where Approximately 80% patients monitored their blood sugar at least once in a 3 months [33]. Patients with less education and family income usually have less doctor visit and hospital attendance, although, they suffer more than their counterparts [32].

In this study, the median travel cost and cost of blood sugar were 30 and 120 BDT respectively. A previous study in Australia reported that cost was not a barrier for access to diabetic services [33]. The majority number of diabetic patients’ travelling distance and time were only 0-5 km and 0-30 min to get to service, however, they had to spend half-day at the hospital. It might happen because of fixed timing for visiting doctor, collecting specimen, overcrowded hospital services and growing number of diabetes patients. A study on utilization of maternal health services in Ethiopia demonstrated similar findings that several barriers of access to health care services, for example, education, age, income, exposer to media as well as family dependency and support [34].

Innovations in creating greater awareness might help to improve diabetes knowledge. In recent years, information technology and mobile phone has reached all segment of the general population in Bangladesh [35, 36]. Mobile phone health (mHealth) programs have shown to improve several disease prevention and management and might be a cost-effective method for improving diabetes knowledge in Bangladesh [37–39].

The findings of this study may not be generalizable to all of Bangladesh as data were collected from only two urban hospitals. It is possible that our study participants had higher levels of education and received better services for diabetes compared to the general diabetic population in Bangladesh. Therefore, the diabetes knowledge, perceptions and service utilization for diabetes care might be lower among the general population with diabetes in other parts of Bangladesh. Other short comings include, purposively sampling, small sample size, short study time and lack of follow up. As self-reported information was collected from the patients, there might be a chance of interviewer bias and recall bias.

## Conclusion

This study shows that diabetes knowledge and utilization of healthcare services for diabetes in urban areas of Bangladesh are affected by socio-demography and socio-economic status of the patients such as, residence, gender, age, level of education, occupation and income. Literacy level, knowledge and perception about T2DM also influenced utilization of services for T2DM. Therefore, innovative and low-costs health behavior changes approaches to increase diabetes knowledge and utilization of healthcare services for diabetes are needed to improve diabetes care in Bangladesh.

## Additional file

Additional file 1: Table S1. Questionnaire. **Figure S1.**Conceptual Framework. **Table S2.**Relationship between SES and Knowledge about Diabetes (*n* = 318). (DOCX 65 kb)

## Abbreviations

AKUH: Aga Khan University Hospital
BIHS: Bangladesh Institute of Health Sciences
CVDs: Cardiovascular Diseases
DMCH: Dhaka Medical College Hospital
HPNSDP: Health Population and Nutrition Sector Development Program
IDF: International Diabetes Federation
LMICs: Low and middle income countries
NCD: Noncommunicable Diseases
SDGs: Sustainable development goals
T2DM: Type 2 Diabetes Mellitus
WHO: World health Organization
