# Supplementary material for: Diabetes knowledge and utilization of healthcare services among patients with type 2 diabetes mellitus in Dhaka, Bangladesh
Source: BMC Health Serv Res. 2017 Aug 22;17:586. doi: 10.1186/s12913-017-2542-3 (PMC5567438; doi:10.1186/s12913-017-2542-3)
Supplement: Additional file 1: Table S1. — Questionnaire. Figure S1.Conceptual Framework. Table S2.Relationship between SES and Knowledge about Diabetes (n = 318). (DOCX 65 kb) [file 12913_2017_2542_MOESM1_ESM.docx]

**Supplementary Table 1. Questionnaire**

| Section A1 : Socio-economic information | | | |
| --- | --- | --- | --- |
| Personal details: *Now I/we would like to collect some personal information about your background* | | | |
| 1.1 | Your age | Years Months | |
| 1.2 | Sex | 1=Male  2=Female |  |
| 1.3 | Religion | 1= Islam  2= Hindu  3= Christian  4= Buddhist  5= Others |  |
| 1.4 | Marital Status | 1= Never married  2= Married  3= Widow/Widower  4= Separated/Divorced |  |
| 1.5 | What is the highest level of education you have completed? | 1=No formal education  2=Less than primary school  3=Primary school completed  4=Secondary school completed  5=Higher secondary school completed  6=College/University completed  7=Post graduate degree completed |  |
| 1.6 | Occupation | 1= Unemployed  2= Service  3= Business  4= Retired  5= Housewife  6= Unable to work  7=Others, please mention in the box |  |
| 1.7 | Participant’s average monthly family expenditure |  |  |
| 1.8 | Total Family Income (monthly average) |  |  |
| 1.9 | How many household members including you live in your house? (eat together) |  |  |
| 1.10 | Area of residence or where do you reside? | 1=Urban  2=Rural |  |

| Section B2: Patients knowledge, perception and access to and utilization of services for Diabetes related questionnaire: | | | |
| --- | --- | --- | --- |
| Sl. | Questions | Responses | Ans. |
| Section B1: Knowledge of prevention, management and complications of Diabetes | | | |
| B2.1 | Do you know the risk factors for Diabetes | 1= Yes  2= No………..skip to D1.3 |  |
| B2.2 | If yes, can you mention about them? | 1= Genetic (family history)  2= Obesity  3= Sedentary Life Style/less physical activity  4= Dietary factors/increased blood sugars  5= Others (diseases, smoking etc.)…………  6= Cannot mention |  |
| B2.3 | Do you know how to prevent diabetes? | 1= Yes  2= No………..skip to D1.5 |  |
| B4.4 | If yes, can you mention about them? | 1= Reduce weight/balanced weight  2= Increased physical activity/walk  3= Balanced Diet (avoid sugar/  carbohydrate, more vegetables)  4= Regular check up  5= Others (treat diseases, stop smoking)………………………………….  6= Cannot mention |  |
| B2.5 | Do you know how to control/manage diabetes? | 1= Yes  2= No………..skip to D1.7 |  |
| B2.6 | If yes, can you mention about them? | 1= Diet  2= Reduce weight  3= Increased physical activity/walking  4= Medication (Oral/SC)  5= Self-management/ regularly check sugar  6= Others (treat diseases, stop smoking)…………………………………  7= Cannot mention |  |
| B2.7 | Do you know the complications of Diabetes | 1= Yes  2= No………..skip to D1.9 |  |
| B2.8 | If yes, can you mention about them? | 1= Uncontrolled diabetics  2= Eye problems (Retinopathy)  3= Kidney problems  4= Neurological problems  5= Cardiovascular problems  6= Others (please specify)……………………..  7= Cannot mention |  |
| B2.9 | Would you like to know more about diabetics? | 1= Yes  2= No |  |
| Section C3:Patients Perception on Diabetes | | | |
| C3.1 | Diabetes is a simple disease | 1=True  2=False  99=Don’t know |  |
| C3.2 | Diabetes is a disease for affluent or rich patients. | 1=True  2=False  99=Don’t know |  |
| C3.3 | Diabetes can cause by excessive intake of sugar. | 1=True  2=False  99=Don’t know |  |
| C3.4 | Diabetes is will of God. | 1=True  2=False  99=Don’t know |  |
| C3.5 | Uncontrolled Diabetes can cause death. | 1=True  2=False  99=Don’t know |  |
| C3.6 | Diabetes is a chronic disease but it can be cured completely. | 1=True  2=False  99=Don’t know |  |
| Section D4: Access & utilization of services related questions | | | |
| D4.1 | Where do you usually go for treatment when you are sick? | 1= MBBS/ Registered Physician  2= Local Health center (GO/NGO/Private)  3= Diabetes hospital/center  4= Homeo /Ayurvedic  5= Village doctor/quack  6= Local Pharmacy  7= Cannot mention |  |
| D4.2 | In the last one year, how many times have you visited a doctor or health center? | Last one year………………………. |  |
| D4.3 | How many times did you visit a doctor in last 3 months? | In last 3 month……………………. |  |
| D4.4 | How far do you have to travel to visit your doctor? | .................Km |  |
| D4.5 | How did you come to this hospital? | 1=On foot  2= Public transport (rickshaw, CNG, bus, train, launch)  3= Private transport (car) |  |
| D4.6 | How long it took to come to this hospital? | .................minutes |  |
| D4.7 | How long do you have to wait to get the service? | .................minutes |  |
| D4.8 | Do you regularly come at this hospital? | 1= Yes  2= No |  |
| D4.9 | How much have you spent to travel here? | ………………. Taka |  |
| D4.10 | How frequently do you check your blood glucose at home? | 1= Several times in a day  2= Daily  3= Weekly  4= Monthly  5= 2-3 monthly  6= Yearly  7= never |  |
| D4.11 | How much did you spend for your blood tests at home during last 3 months? | In last 3 month................ |  |
| D4.12 | Who pays for all your medical costs? | 1= Self  2= A relative or friend  3= Self or private insurance  4= Government insurance  5= Employer |  |
| D4.13 | How satisfied are you with the support you get from family and friends for dealing with your diabetes? | 1= Very satisfied  2= Satisfied  3= Little satisfied  4= Dissatisfied  5= Very dissatisfied |  |
| D4.14 | How satisfied are you with the support you get from your health care team for dealing with your diabetes? | 1= Very satisfied  2= Satisfied  3= Little satisfied  4= Dissatisfied  5= Very dissatisfied |  |
| D4.15 | Would you recommend your relatives to have diabetes services at this hospital? | 1= Yes  2= No  89= Others (please specify)…………………….. |  |
| D4.16 | How satisfied are you with your current health care experience? | 1= Very satisfied  2= Satisfied  3= Little satisfied  4= Dissatisfied  5= Very dissatisfied |  |
| The End | | | |

**Supplementary Table 2 Relationship between SES and Knowledge about Diabetes (n = 318).**

|  | Socio-demographic variables | Knowledge (%) | | | Statistical Indices |
| --- | --- | --- | --- | --- | --- |
|  |  | Good | Average | Poor |  |
| Gender | Male | 27 (20.1) | 86 (64.2) | 21 (15.7) | ᵡ^2=12.505^  df=2  P=0.002 |
|  | Female | 14 (7.6) | 125 (67.9) | 45 (24.5) |  |
| Age | 30-39 | 13 (28.6) | 27 (60.0) | 5 (11.4) | ᵡ^2=4.792^  df=6  P=0.571 |
|  | 40-49 | 14 (16.5) | 56 (65.9) | 15 (17.6) |  |
|  | 50-59 | 8 (8.5) | 67 (64.9) | 19 (20.2) |  |
|  | ≥60 | 6 (6.4) | 61 (64.9) | 27 (28.7) |  |
| Education | No formal education | 2 (10.4) | 17 (44.6) | 19 (50.0) | ᵡ^2=49.007^  df=6  P=0.000 |
|  | Primary | 7 (6.8) | 71 (68.9) | 25 (24.3) |  |
|  | Secondary | 11 (10.2) | 82 (75.9) | 15 (13.9) |  |
|  | College & above | 21 (30.4) | 41 (59.5) | 7 (10.1) |  |
| Occupation | Service | 10 (18.5) | 38 (70.4) | 6 (11.1) | ᵡ^2=25.312^  df=12  P=0.013 |
|  | Business | 8 (25) | 19 (59.4) | 5 (15.6) |  |
|  | Laborer | 0 (0) | 6 (85.7) | 1 (14.3) |  |
|  | Farming | 0 (0) | 3 (60) | 2 (40) |  |
|  | Housewife | 43 (24.4) | 120 (68.2) | 13 (7.4) |  |
|  | Retired | 10 (24.4) | 24 (58.5) | 7 (17.1) |  |
|  | Others | 0 (0) | 1 (33.3) | 2 (66.7) |  |
| Family Income | < 10,000 BDT | 0 (0) | 10 (38.5) | 16 (61.5) | ᵡ^2=40.712^  df=8  P=0.000 |
|  | 10,000-29,999 BDT | 16 (11.5) | 95 (68.4) | 28 (20.1) |  |
|  | 30,000-59,000 BDT | 12 (11.7) | 76 (73.7) | 15 (14.6) |  |
|  | 60000-89,000 BDT | 8 (33.3) | 13 (54.2) | 3 (12.5) |  |
|  | ≥ 90000 BDT | 5 (19.2) | 17 (65.4) | 4 (15.4) |  |
| Residence | Urban | 32 (13.7) | 157 (67.1) | 45 (19.2) | ᵡ^2=4.613^  df=4  P=0.329 |
|  | Rural | 4 (10.3) | 22 (56.4) | 13 (33.3) |  |
|  | Semi-urban | 5 (11.1) | 32 (71.1) | 8 (17.8) |  |

BDT Bangladeshi Taka (1 USD=80 BDT, 2015).

**Supplementary Figure 1. Conceptual Framework**
